# Early-onset parkinsonism in a pedigree with phosphoglycerate kinase deficiency and a heterozygous carrier: do *PGK-1* mutations contribute to vulnerability to parkinsonism?

**DOI:** 10.1038/s41531-017-0014-4

**Published:** 2017-03-31

**Authors:** Satoshi Sakaue, Takashi Kasai, Ikuko Mizuta, Masaya Suematsu, Shinya Osone, Yumiko Azuma, Toshihiko Imamura, Takahiko Tokuda, Hitoshi Kanno, Omar M. A. El-Agnaf, Masafumi Morimoto, Masanori Nakagawa, Hajime Hosoi, Toshiki Mizuno

**Affiliations:** 1 Department of Pediatrics, Graduate School of Medical Science, Kyoto Prefectural University of Medicine, Kyoto, 602-8566 Japan; 2 Department of Neurology, Graduate School of Medical Science, Kyoto Prefectural University of Medicine, Kyoto, 602-8566 Japan; 3 AMED-CREST, Japan Agency for Medical Research and Development, Kyoto, 602-8566 Japan; 4 Department of Molecular Pathobiology of the Brain Diseases, Graduate School of Medical Science, Kyoto Prefectural University of Medicine, Kyoto, 602-8566 Japan; 5 Department of Transfusion Medicine and Cell Processing, Tokyo Women’s Medical University, Tokyo, 162-8666 Japan; 6 Neurological Disorders Center, Qatar Biomedical Research Institute (QBRI), and College of Science and Engineering, Hamad Bin Khalifa University (HBKU), Education City, Qatar Foundation, Doha P.O. Box 5825, Doha, Qatar; 7 North Medical Center, Graduate School of Medical Science, Kyoto Prefectural University of Medicine, Kyoto, 602-8566 Japan

## Abstract

Phosphoglycerate kinase 1 (PGK-1) is a glycolytic enzyme encoded by *PGK-1*, which maps to the X chromosome. PGK-1 deficiency causes X-linked recessive hereditary chronic hemolytic anemia, myopathy, and neurological disorders due to insufficient ATP regeneration. Early-onset parkinsonism has occasionally been reported as a neurological complication of this condition. However, heterozygous carriers of PGK-1 deficiency were thought to be neurologically asymptomatic. Here, we report a boy with PGK-1 deficiency and his mother, a carrier of a heterozygous mutation in *PGK-1*, both of whom presented with early-onset parkinsonism. The boy developed parkinsonism at 9 years of age. His parkinsonism partially responded to levodopa treatment. ^123^l-metaiodobenzylguanidine (MIBG) uptake was normal. His mother, who exhibited normal PGK-1 activity in erythrocytes, developed parkinsonism at 36 years of age. Her symptoms were undistinguishable from those of Parkinson’s disease (PD), despite her normal uptake of MIBG. Neither a point mutation in nor multiplication of *SNCA* was found. Additionally, hotspots of *LRRK2* and *GBA* were not mutated. To our knowledge, this report provides the first description of parkinsonism in a carrier of PGK-1 deficiency. Interestingly, *PGK-1* is located within the confirmed susceptibility locus for PD known as *PARK12*. These observations suggest that *PGK-1* mutations confer susceptibility to PD.

## Early-onset Parkinson’s disease: a new genetic link

Mutations in the gene encoding phosphoglycerate kinase 1 (PGK-1) may confer susceptibility to early-onset Parkinson’s disease (PD). PGK-1 is a crucial protein for the breakdown of sugar in the body and mutations that cause PGK-1 deficiency lead to an X-linked metabolic disorder characterised by the breakdown of red blood cells, muscle weakness and various central nervous system abnormalities. Takashi Kasai at Kyoto Prefectural University of Medicine, Japan, and colleagues describe early-onset PD symptoms in a 9 year old boy with PGK-1 deficiency and his mother at 36 years of age. This is the first report of parkinsonism developing in an otherwise asymptomatic carrier of a PGK-1 mutation. The location of the PGK-1 gene on the X chromosome is within a confirmed susceptibility region for PD known as PARK12, suggesting that PGK-1 may directly contribute to the disease.

## Introduction

Phosphoglycerate kinase 1 (PGK-1) is a key enzyme in the glycolytic pathway and is encoded by *PGK-1* (Online Mendelian Inheritance in Man (OMIM) #311800), which maps to the X chromosome and is expressed in all somatic tissues. PGK-1 deficiency (OMIM #300653) is an uncommon cause of congenital nonspherocytic hemolytic anemia. Clinical features of this deficiency include X-linked recessive chronic hemolytic anemia, myopathy and neurological disorders (e.g., seizures and mental retardation). These phenotypes are diverse among patients due to the differential involvement of three components: erythrocytes; skeletal muscle; and the central nervous system.^1^ Parkinsonism occasionally accompanies PGK-1 deficiency in males.^2, 3^ In contrast, heterozygous carriers of this deficiency are generally thought to be asymptomatic. Here, we report a boy with PGK-1 deficiency and his mother, a heterozygous carrier of a *PGK-1*mutation, both of whom presented with early-onset parkinsonism.

### Cases

**Fig. 1:**
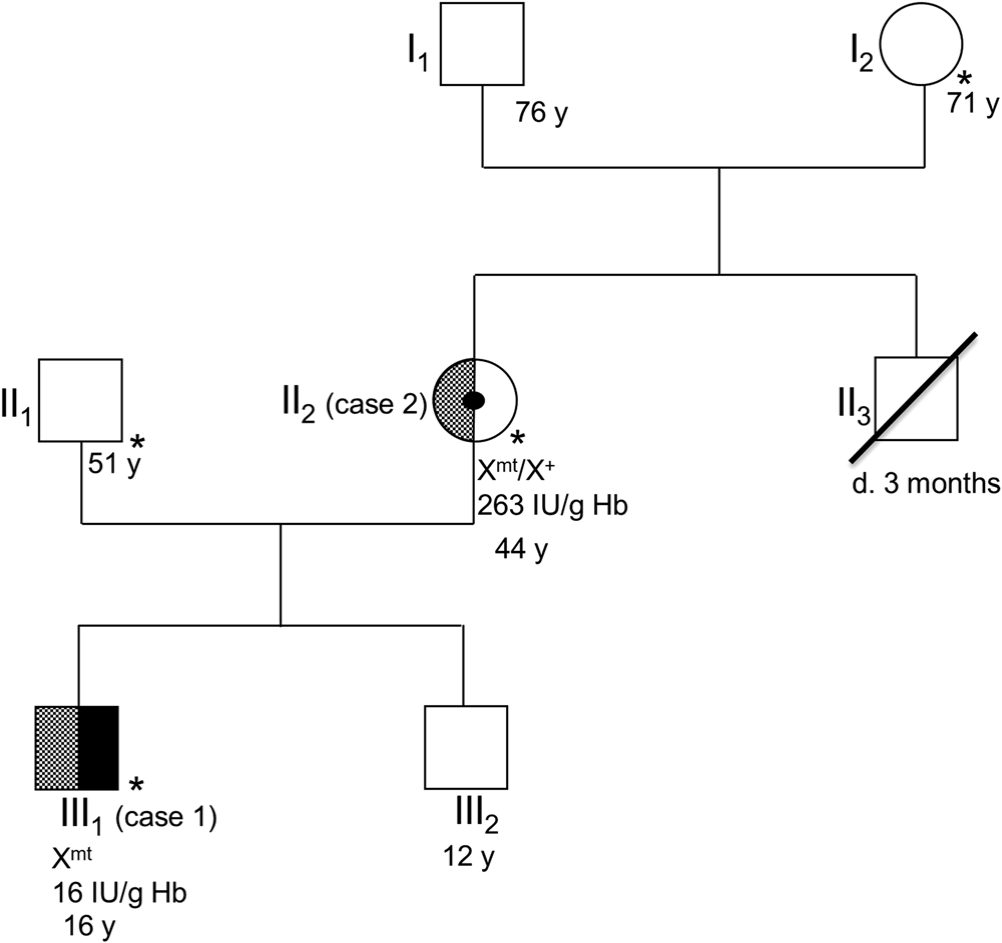
The patient’s pedigree is presented in accordance with standardized human pedigree nomenclature. *Solid black* indicates the phenotype of classical symptoms of PGK-1 deficiency (i.e., hemolytic anemia and myopathy). The *checkerboard pattern* indicates the phenotype of parkinsonism. Members with *asterisks* were neurologically examined by the authors. Cases 1 (III_1_) and 2 (II_2_) were also genetically and enzymatically examined. The results of genetic and enzymatic testing for PGK-1 are presented below these cases. X^mt^ and X^+^ indicate an allele with the c.1060G>C mutation and the wild-type allele on the X chromosome, respectively

The examined family comes from western Japan and includes six living members in three generations, with no history of consanguinity (Fig. 1).

#### Case 1

**Fig. 2:**
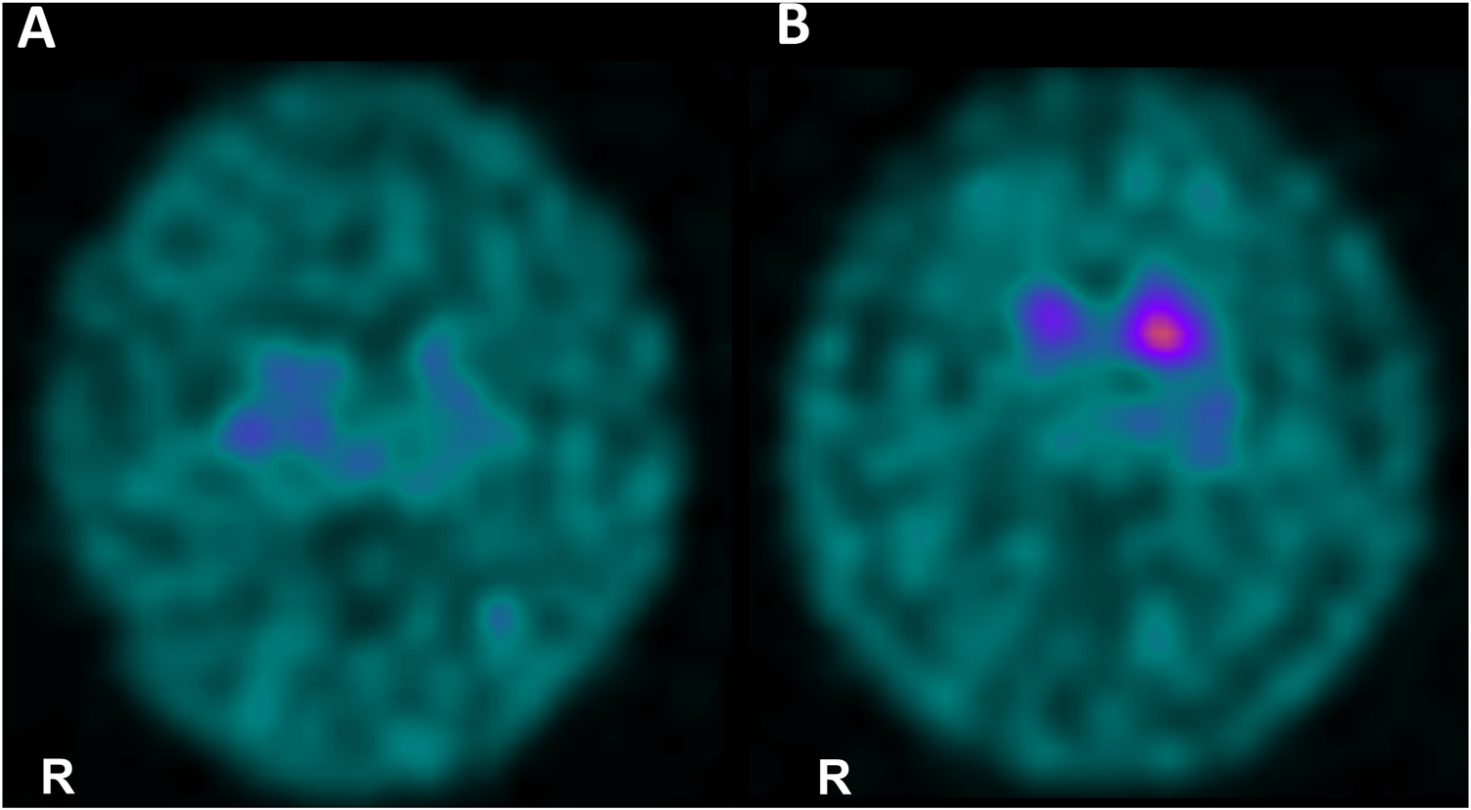
DAT images for case 1 (**a**) and case 2 (**b**) are presented. Both images reveal decreased DAT uptake in the striatum. Specific binding ratios were semiquantitatively calculated using DaT View software (Nihon Medi-Physics, Tokyo, Japan) based on Bolt’s method.^10^ The right and left specific binding ratios were 1.87 and 1.71, respectively, in (**a**) and 2.50 and 4.19, respectively, in (**b**). The reported cut-off value was 4.5.^10^

(III_1_ in Fig. 1). A 16-year-old boy with PGK-1 deficiency developed parkinsonism. We previously reported that this patient was diagnosed with PGK-1 deficiency at 3 years of age based on decreased PGK activity in erythrocytes (16 IU/g Hb, normal: 255–325 IU/g Hb) and the novel *PGK-1* missense mutation c.1060G > C; p.A354P.^4^ (Notably, this mutation was originally described as A353P, which indicates an amino-acid substitution at the 353rd position from the NH_2_-terminal serine residue.^4^). He had been repeatedly hospitalized for recurrent episodes of myoglobinuria with hemolytic anemia every few months. Moderate intellectual disability had been identified prior to 3 years of age. No epileptic seizures were observed. At 9 years of age, the patient presented with action tremor in his extremities. Rigidity developed at 11 years of age. Therapy with carbidopa/levodopa 25/100 mg three times daily produced immediate but partial improvement in the patient’s parkinsonism. However, his symptoms gradually progressed. When the patient was 16 years of age, neurological examination revealed severe action tremor in his extremities, especially his left arm, and a mask-like face. Marked rigidity with a dystonic posture was observed in all limbs and the neck. He could no longer walk without assistance. He exhibited both urinary and fecal incontinence but no orthostatic hypotension. Brain magnetic resonance imaging revealed mild atrophy of the cerebellum and the basis pontis. Neither the hot cross bun sign nor the putaminal slit sign was identified (Supplementary Fig. 1). A dopamine transporter (DAT) scan using ^123^I-Ioflupane revealed markedly reduced striatal DAT binding (Fig. 2a). Myocardial imaging with ^123^l-metaiodobenzylguanidine (MIBG) was normal (heart-to-mediastinum ratio for delayed image: 3.08, institutional normal range: 1.97 to 3.75).

#### Case 2

(II_2_ in Fig. 1). The mother of case 1 (initially examined for this study at 44 years of age) was a known carrier of the same heterozygous mutation in *PGK-1*.^4^ At the age of 36 years, she developed resting tremor and a short-stepped gait. Her prior medical history was unremarkable, and she did not have hemolytic anemia or neurologic disorders. PGK-1 activity in her erythrocytes was within the normal range (263 IU/g Hb). Neurological examination revealed rigidity in the left upper extremity and both lower extremities, a mask-like face and resting tremor. She had mild fecal incontinence. Neither urinary incontinence nor orthostatic hypotension was observed. Brain computed tomography produced normal findings. Carbidopa/levodopa 25/100 mg three times daily resulted in symptomatic improvement. When the patient was 45 years of age, a DAT scan revealed a right-dominant dopaminergic deficit in the striatum (Fig. 2b).^123^l-MIBG myocardial imaging was normal (heart-to-mediastinum ratio for delayed image = 2.36).

As indicated in Fig. 1, neither a history of hemolytic anemia nor neurological dysfunction, including Parkinson’s disease (PD), was identified in the family except for cases 1 and 2. II_3_, a brother of II_2_, died due to cardiac disease at 3 months of age.

To exclude known causes of autosomal dominant parkinsonism, we performed genetic analyses of the coding region and gene dose of *SNCA*, the hotspot of *GBA* (exons 5, 6, 8, and 10, including R120W and L44P/RecNciI),^5^ and the hotspot of *LRRK2* (exons 31, 35, 41, and 48, including R1441G/H/C, Y1699C, G2019S, I2020T and G2385R).^6^ No mutation was found in either of the aforementioned cases.

## Discussion

Here, we describe a boy with PGK-1 deficiency and his mother, a heterozygous carrier of a *PGK-1* mutation, both of whom presented with early-onset parkinsonism. It is unknown whether II_3_ died from PGK-1 deficiency. Unfortunately, we could not obtain consent for a genetic test from the maternal grandmother of case 1 (I_2_), who appeared to be neurologically normal. Therefore, we have no information regarding whether the mutation in case 2 was inherited from the patient’s mother or was a de novo mutation. Neurological symptoms of the boy in case 1 included parkinsonism and were accompanied by cerebellar atrophy, autonomic dysfunction, and normal MIBG uptake. These symptoms may be similar to those of multiple system atrophy-parkinsonism rather than PD. Symptoms exhibited by this boy’s mother included levodopa-responsive parkinsonism with resting tremor and were consistent with clinical diagnostic criteria for PD, despite her normal uptake of MIBG. To our knowledge, this report provides the first evidence that parkinsonism can develop not only in a patient with PGK-1 deficiency but also in a heterozygous carrier of a *PGK-1* mutation without an enzymatic deficiency. We did not comprehensively exclude all known genetic abnormalities related to susceptibility to parkinsonian syndromes, including PD. However, our cases appear unlikely to be caused by known parkinsonism-related mutations for the following reasons. First, parkinsonism that develops in patients under the age of 10 years is quite unusual for known dominantly inherited forms of PD. Second, in our cases, parkinsonism was more severe and exhibited an earlier age of onset in the child compared with the mother. The appearance of more severe symptoms at earlier ages in successive generations has not been reported in known hereditary parkinsonian syndromes, whereas this phenomenon is easy to understand when we consider differences in gene dosages of *PGK-1*between males and females.

According to a current database (OMIM #311800), *PGK-1* lies on the X chromosome at 78,065,188–78,129,296, a region within Xq21.1, although *PGK-1* was originally reported to map to chromosome Xq13.^7^ Interestingly, the region between Xq21 and q25 is known as a susceptibility locus for classical PD associated with *PARK12* (OMIM #300557), and the causative gene for this disease has not been identified.^8, 9^ We therefore speculate that mutations in *PGK-1* may contribute to the pathogenesis of *PARK12*-associated PD*.*

Parkinsonism in patients with PGK-1 deficiency has been postulated to occur due to insufficient ATP regeneration in the substantia nigra as a result of low levels of PGK activity.^2^ In heterozygous carriers, the mutant allele of *PGK-1* on the X chromosome is randomly inactivated during early embryonic stages due to lyonization, resulting in a “mosaic” or “patchy” pattern of enzymatic activity. Therefore, selective enzymatic deficiency in the substantia nigra is possible in heterozygous carriers even when erythrocytes exhibit normal enzymatic activity, as observed in the present case. Based on this hypothesis, the penetrance of parkinsonism in PGK-1 deficiency is expected to be incomplete since variability in lyonization would lead to considerable variability in the severity of parkinsonism in female carriers.

Several studies have established an association between heterozygous mutations in *GBA*, which is responsible for Gaucher disease, and PD. These findings have induced a paradigm shift in the PD field from the “common disease: common variant” hypothesis to the “common disease: multiple rare variant” hypothesis.^5^ We may have encountered a similar situation in this family. Neurologists and pediatricians should therefore look carefully for parkinsonism in not only PGK-1 deficiency patients but also carriers of this deficiency. Further studies are needed to clarify whether multiple rare variants of *PGK-1* confer susceptibility to PD or other parkinsonian syndromes.

## Electronic supplementary material

Supplemental Figure Legend

Supplemental Figure
