# Supplementary material for: Early-onset parkinsonism in a pedigree with phosphoglycerate kinase deficiency and a heterozygous carrier: do PGK-1 mutations contribute to vulnerability to parkinsonism?
Source: NPJ Parkinsons Dis. 2017 Mar 31;3:13. doi: 10.1038/s41531-017-0014-4 (PMC5459803; doi:10.1038/s41531-017-0014-4)
Supplement: Supplementary file 1 — Supplemental Figure Legend [file 41531_2017_14_MOESM1_ESM.doc]

Case Report

Early-onset parkinsonism in a pedigree with phosphoglycerate kinase deficiency and a heterozygous carrier: Do *PGK-1* mutations contribute to vulnerability to parkinsonism?

Supplementary Figure Legend

T2-weighted magnetic resonance images for case 1 showing slight shrinkage of the basis pontis with mild atrophy of the cerebellum.
