# Supplementary figures and images for: Early-onset parkinsonism in a pedigree with phosphoglycerate kinase deficiency and a heterozygous carrier: do PGK-1 mutations contribute to vulnerability to parkinsonism?
Source: NPJ Parkinsons Dis. 2017 Mar 31;3:13. doi: 10.1038/s41531-017-0014-4 (PMC5459803; doi:10.1038/s41531-017-0014-4)

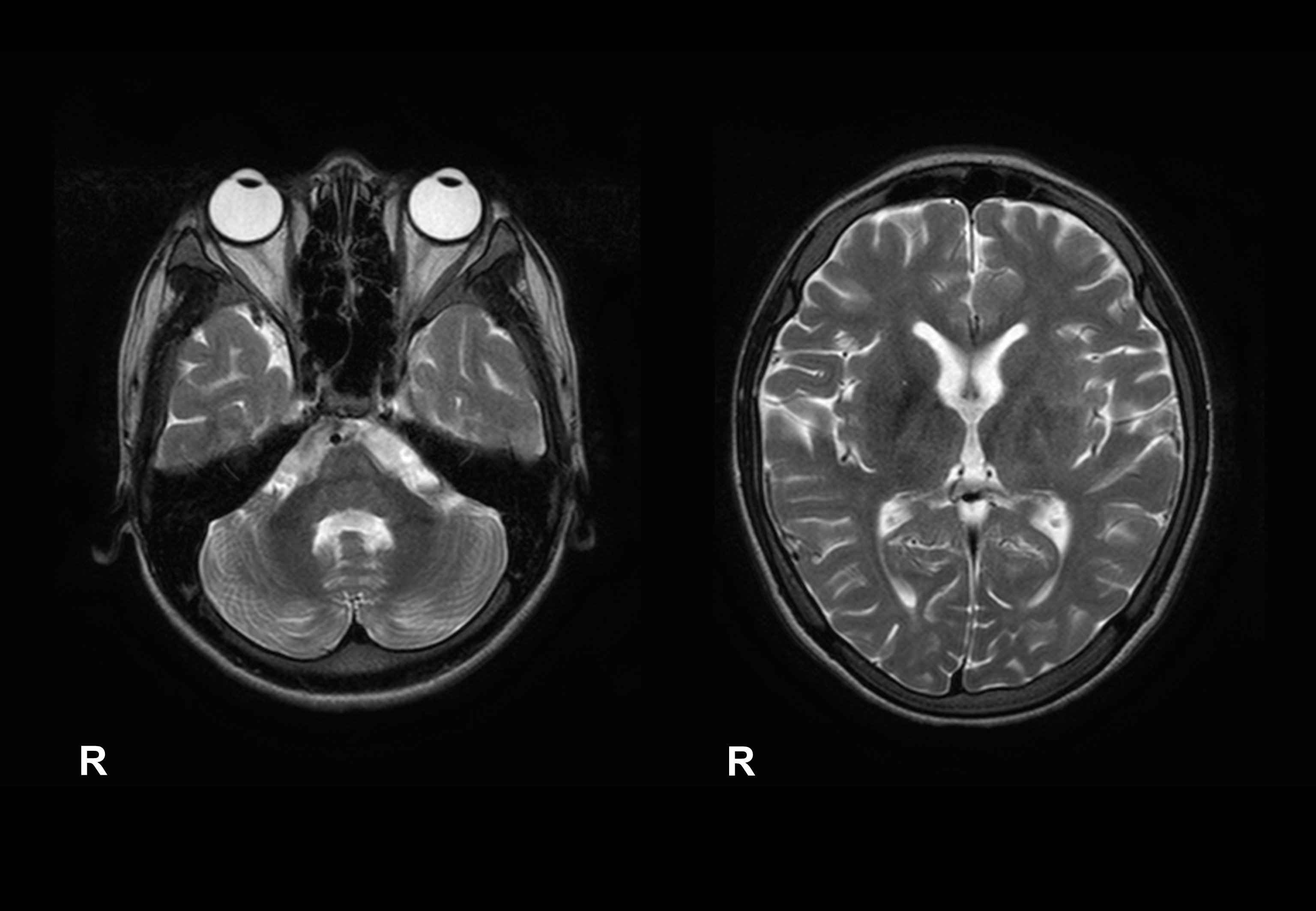

Supplement: Supplementary file 2 — Supplemental Figure [file 41531_2017_14_MOESM2_ESM.tif]
